# Multifaceted antifungal mechanisms of volatile organic compounds emitted from *Pseudomonas chlororaphis* ZL3 against *Botrytis cinerea*

**DOI:** 10.1128/spectrum.02706-25

**Published:** 2025-11-24

**Authors:** Chunwei Wang, Yaxin Su, Wenyan Miao, Ziyu Jin, Tiankun Duan, Meiqin Wang, Yan Wang

**Affiliations:** 1 Shanxi Key Laboratory of Bioagent Utilization and Eco-Pesticide Innovation, College of Plant Protection, Shanxi Agricultural University 74600, Taigu, Shanxi, PR China; USDA-ARS San Joaquin Valley Agricultural Sciences Center, Parlier, California, USA

**Keywords:** *Pseudomonas chlororaphis*, volatile organic compounds, antifungal mechanism, *Botrytis cinerea*, transcriptomics, metabolomics

## Abstract

To develop an effective approach to inhibit *Botrytis cinerea*, a new biocontrol strain, *Pseudomonas chlororaphis* ZL3, was screened and exhibited excellent biocontrol potential. Nevertheless, the antifungal mechanism of *P. chlororaphis* volatile organic compounds (VOCs) against *B. cinerea* is still unclear. In this study, VOCs emitted from *P. chlororaphis* ZL3 were found to have potent inhibitory effects on mycelium growth, conidia germination, and sclerotium formation. Subsequently, the antifungal mechanism was revealed via the biophysical, transcriptomic, and metabolomic analysis. Compared to the control groups, the relative conductivities increased dramatically, while the ergosterol content decreased sharply, determining that VOCs could enhance cell membrane permeability and destroy cell membrane integrity. Additionally, VOCs could also inhibit ATPase activity and reduce reactive oxygen species (ROS) accumulation, thereby adversely interfering with energy metabolism. The superoxide dismutase (SOD) and catalase (CAT) activities were significantly reduced in *B. cinerea* mycelia, presenting the impairment of the antioxidant defense mechanism. Furthermore, most of the differential expression genes (DEGs) were involved in lipid metabolism, membrane transport, energy production, and conversion, indicating that the VOCs might affect the structure and function of cell membranes and energy metabolism. The differential metabolites were mainly involved in the alteration of amino acid metabolism and lipid metabolism. Together, these findings provide valuable insights into the molecular responses of *B. cinerea* to *P. chlororaphis* ZL3 VOCs and support the potential development of novel biological fumigants.

**IMPORTANCE:**

Biological control is a sustainable alternative way for the control of gray mold. The volatile organic compounds emitted from *Pseudomonas chlororaphis* ZL3 had high inhibitory activity against *Botrytis cinerea*, but the antifungal mechanism is largely unknown. This study attempted to interpret the comprehensive antifungal mechanism, providing a theoretical foundation for the application of volatile organic compound (VOC)-mediated biological fumigants in the future.

## INTRODUCTION

As one of the most destructive plant pathogens, *Botrytis cinerea* could infect more than 1,400 plant species (belonging to 600 plant genera), including vegetables, fruits, and ornamentals, during the growth and postharvest storage period (1, 2). *B. cinerea*, a necrotrophic fungal pathogen, causes large masses of gray conidia known as gray mold and was found to be associated with various diseases, such as root rot, blossom blight, leaf spot, fruit decay, and stem canker worldwide (3, 4). Due to its short life cycle, broad host range, and high sporulation ability, *B. cinerea* is usually involved in perishable fresh agricultural food and has proved to be responsible for considerable economic losses of more than €1 billion per annum (1, 3).

To avoid the risks of severe damage, chemical controls have been widely applied to prevent *B. cinerea* infection and reduce the microbial dissemination (1). Currently, chemical fungicides have always been used to suppress the occurrence of gray mold in the field (5). However, frequent use of chemicals has high risks to cause environmental pollution, ecosystem destruction, animal and human health hazards, and pathogen resistance (6, 7). It is a difficult issue to reduce the consumption of chemicals worldwide (8). Hence, it is of great urgency to develop non-chemical strategy alternatives to chemical in the controlling of gray mold.

Recently, biological control might be regarded as a sustainable alternative way for controlling gray mold because of the high antifungal ability, ecological security, and environmental compatibility (7, 9, 10). A lot of antagonistic microorganisms have the potential to control crop gray mold (11–13). Among these antagonistic microorganisms, *Pseudomonas* species could play a critical role in the management of gray mold based on the various mechanisms, including inhibition of pathogen growth, production of antimicrobial substances, and induction of plant resistance (8, 14–16).

Nowadays, microbial volatile organic compounds (mVOCs), the odorous molecules with low-molecular-weight (<300 Da), are found to be easily dispersed through air and water and have attracted great attention in the inhibition of fungal growth and disease development (9, 17). Moreover, the microbial VOCs have excellent biocontrol efficacy, remain little residual risk on the surface of crops, and might be classified as important beneficial biocontrol agents for the suppression of gray mold (8, 9, 12). The microbial VOCs were also found to have multiple advantages, including less pollution to the environment, low toxicity, and high antifungal activities (18). It is generally acknowledged that microbial VOCs could play essential roles in inhibiting *B. cinerea,* causing gray mold, and have increasingly gained a lot of attention worldwide (8, 19). Previous works also demonstrated that *Pseudomonas* species can emit antifungal VOCs (8, 20–22). In our prior work, a new biocontrol strain, *Pseudomonas chlororaphis* ZL3, was isolated from Chinese cherry fruit (19). Nevertheless, to our knowledge, antifungal mechanisms of the VOCs from *P. chlororaphis* against *B. cinerea* still remain largely unknown.

Multi-omics, including transcriptomics and metabolomics, has proven to be an effective approach to understanding the gene expression level and metabolic accumulation of the plant pathogens treated with antagonistic microorganisms over the last decade (7, 23, 24). Transcriptomics technology has been extensively used in revealing the antifungal mechanisms of biocontrol strains against plant pathogens at the molecular level (7). Metabolomics, regarded as an advanced omics technology, has been applied to comprehensively analyze the metabolites in biochemical processes between beneficial and pathogenic microorganisms (25). In this work, an integrated analysis of transcriptomics and metabolomics will be conducted to understand the antifungal molecular mechanism of *P. chlororaphis* ZL VOCs.

Herein, this work aimed to comprehensively elucidate the systematic antifungal mechanism of VOCs from *P. chlororaphis* ZL3 against *B. cinerea* based on biophysical, transcriptomic, and metabolomic levels, which will be beneficial to interpret the molecular responses of *B. cinerea* to the VOCs and would be helpful for the exploitation of VOC-mediated biological fumigants.

## MATERIALS AND METHODS

### Plant pathogen and biocontrol strain

*B. cinerea* isolate YT1 was isolated from the postharvest gray mold on Chinese Cherry using the tissue isolation method and identified based on its morphological characteristics and internal transcribed spacer and DNA-dependent RNA polymerase subunit II (RPB2). Strain YT1 was stored on potato dextrose agar (PDA) medium at 4°C. *P. chlororaphis* strain ZL3, originally isolated from Chinese cherry fruit, was stored at −80°C and transferred into Luria-Bertani (LB) medium before use (19). All the strains in this work were maintained at 4°C.

### Antifungal effects of *P. chlororaphis* ZL3 VOCs on *B. cinerea*

The effects of *P. chlororaphis* ZL3 VOCs on mycelium growth, conidium germination, and sclerotium formation of *B. cinerea* were investigated using a double Petri-dish assay previously reported by Alijani et al. (26) and Wang et al. (9). Each treatment was replicated three times. Bacterial suspension was prepared in sterile LB medium at a concentration of 10^7^ colony-forming units (CFU). Subsequently, 100 µL of the suspension was added to a nutrient agar (NA) plate. Correspondingly, a *B. cinerea* plug (6 mm in diameter) was transferred to the center of the PDA plate. Subsequently, the tested NA and PDA plates were tightly sealed with parafilm and then incubated at 25°C. The NA plate without bacterial suspension was used as a control. The colony diameters of each treatment were measured at 1, 2, 3, and 4 days after inoculation (9). The inhibition rates were determined according to the formula (C_d_ − T_d_) × 100%/(C_d_ − C_0_), where C_d_ and T_d_ represent the colony diameter of the control and treatment, respectively, and C_0_ represents the diameter of the mycelial plug (18). To determine the inhibition effects of the VOCs on sclerotium formation, the sclerotium quantity and weight were measured at 20 days after inoculation.

For the evaluation of the effects of VOCs on the conidium germination, 100 µL of conidium suspension (10^6^ conidia/mL) was inoculated on a water agar (WA) plate, whereas 100 µL of bacterial suspension was placed on an NA plate. Next, the tested WA and NA plates were tightly sealed with parafilm. After incubation at 25°C for 6 h, the conidium germination percentages were calculated based on the observation of 100 conidia (27). Moreover, the mycelium morphology of *B. cinerea* was further observed under the stress of VOCs (9). The morphological changes of *B. cinerea* were examined using a JSM-6490LV scanning electron microscopy (SEM, JEOL Ltd., Japan).

### Effects of *P. chlororaphis* ZL3 VOCs on mycelia plasma membrane

Mycelium plasma membrane was investigated as described by Ashraf and Ali (28) and Duan et al. (29). Firstly, a *B. cinerea* plug (6 mm in diameter) was transferred into 100 mL of PDB medium. After incubation at 180 r/min and 25°C for 2 days, *B. cinerea* mycelia were filtered through sterile gauze and washed with 1 mM phosphate-buffered saline (PBS) solution (pH 7.4). Next, the tested mycelia (0.5 g) was placed in 100 mL of sterile water. Above the liquid surface, a sterile lid containing 100 µL of the bacterial inoculum (1 × 10^6^ CFU/mL) was used to treat the mycelia. The mycelium cultures were treated with sterile water as controls. After incubation at 180 r/min and 25°C for 2, 4, 6, 8, and 16 h, the conductivity of *B. cinerea* mycelium was assessed by a DDSJ-308A conductivity meter (Shanghai INESA Scientific Instrument Co., Ltd., China). Relative conductivity was calculated as Relative conductivity % = (L − L_0_)/(L_1_ − L_0_), where L is the conductivity at different intervals, L_0_ is the initial conductivity, and L_1_ represents the conductivity of mycelia autoclaved at 121°C for 20 min. Each treatment was replicated three times.

The ergosterol content was also estimated using the modified method (30). Briefly, *B. cinerea* mycelia were treated using a double Petri-dish assay as described in Section “Antifungal effects of *P. chlororaphis* ZL3 VOCs on *B. cinerea*” and collected at 4, 5, and 6 days after treatment with *P. chlororaphis* ZL3 VOCs. *B. cinerea* mycelia were washed with sterile water for wet weight determination. Next, the tested sample was resuspended in potassium hydroxide ethanol solution, vortexed for 2 min, and then treated at 85°C for 4 h. Then, 1 mL of sterile distilled water and 3 mL of heptane were added to extract the ergosterol and incubated for 1 h. Lastly, the absorbance of the heptane phase at 230 and 282 nm was determined using a UV-Spectrophotometer. The ergosterol content was calculated based on the formula described by Yue et al. (8): Ergosterol (%) = (A_282_/290)/weight − (A_230_/518)/weight.

### Effects of *P. chlororaphis* ZL3 VOCs on protein concentration, ATPase, SOD, CAT, PG, β-1, 3-GA, and CHI activities

A 6 mm mycelial plug was transferred into 100 mL of PDB medium and shaken at 180 r/min at 25°C for 36 h. Next, a sterile lid containing 100 µL of bacterial inoculum (1 × 10^6^ CFU/mL) was suspended above the liquid surface. After shaking at 180 r/min at 25°C for 1, 2, 3, 4, and 5 days, *B. cinerea* mycelia were filtered through sterile gauze. Next, superoxide dismutase (SOD), catalase (CAT), polygalacturonase (PG), β-1,3-glucanase (GA), and chitinase (CHI) activities were measured using the corresponding SOD (catalog no. BC0175), CAT (catalog no. BC0205), PG (catalog no. BC2665), GA (catalog no. BC0365), and CHI (catalog no. BC0825) activity assay kits (Solarbio Life Sciences, Beijing, China). *B. cinerea* mycelia were also treated using a double Petri-dish assay as described in Section “Antifungal effects of *P. chlororaphis* ZL3 VOCs on *B. cinerea.*” Total protein concentration was determined using the biuret method (31). ATPase was detected using available kits (Beijing Solarbio Science & Technology Co., Ltd., China).

### Effects of VOCs from *P. chlororaphis* ZL3 on DNA content and ROS accumulation

After treatment with *P. chlororaphis* ZL3 VOCs for 5 days, the DNA contents and reactive oxygen species (ROS) levels in *B. cinerea* mycelia were measured using the method described by Ma et al. (32) and Duan et al. (29) with some modifications. Acridine orange (AO) was considered a biological fluorescent probe for the assessment of DNA content (33). The fluorescent dye 2′,7′-dichlorofluorescein diacetate (DCFH-DA) was widely used to determine ROS accumulation (29, 32). The fungal mycelia collected from the VOCs treatments and control groups were stained with 0.1% AO and 5 µg/mL DCFH-DA dye in the dark and incubated at 30 and 60 min, respectively. Subsequently, the stained mycelia were washed with PBS three times. The effects of the VOCs on DNA content and ROS levels were determined using a TCS SP8 confocal laser scanning microscope (CLSM, Leica, Mannheim, Germany).

### Transcriptome analysis

A *B. cinerea* mycelial disc was placed onto a PDA plate. The PDA plate was treated with 100 µL of *P. chlororaphis* ZL3 bacterial inoculum (1 × 10^6^ CFU/mL) using a double Petri-dish assay as described in Section “Antifungal effects of *P. chlororaphis* ZL3 VOCs on *B. cinerea.*” A 100 µL of sterile LB medium was used as a control. After incubation at 23°C for 4 days, 100 mg of mycelia were collected from PDA cultures, placed into a 1.5 mL tube, followed by treatment with liquid nitrogen. Next, total RNA was subsequently extracted using TRIzol Reagent (Invitrogen, California, USA). Subsequently, 1 µg of total RNA was used to construct an RNA-seq transcriptome library using Illumina Stranded mRNA Prep, Ligation (San Diego, California, USA), and then sequenced using NovaSeq Reagent Kit (Illumina, San Diego, California, USA) at the NovaSeq 6000 platform.

Differential expression analysis was performed by DESeq2 software (23). Next, all differential expression genes (DEGs) were subjected to the Gene Ontology (GO) database for GO enrichment analysis (8). Furthermore, Kyoto Encyclopedia of Genes and Genomes (KEGG) pathway analysis was performed by Python scipy software. The quantitative reverse transcription polymerase chain reaction (qRT-PCR) method was used to evaluate the gene expression of eight DEGs. The qRT-PCR primer sets are shown in Table S1. The experiment for qRT-PCR shared the same total RNA as the transcriptome analysis above. The gene expression levels were calculated using the 2^−ΔΔCT^ method described by Schmittgen and Livak (34). Each experiment contained three biological replicates.

### Metabolomic analysis

*B. cinerea* mycelia were prepared for metabolomic analysis. Firstly, 100 mg of fungal mycelia and 400 µL of methanol: water (4:1, vol/vol) solution were added to a 2 mL centrifuge tube. L-2-chlorophenylalanine (0.02 mg/mL) was added as an internal standard. Next, the mixture was settled at −10°C and ground by using a Wonbio-96c high-throughput tissue crusher at 50 Hz for 6 min. Subsequently, the sample was allowed for ultrasound at 40 kHz for 30 min at 5°C, placed at −20°C for 30 min, and centrifuged at 13,000 *× g* at 4°C for 15 min. The supernatant was used for further metabonomic analysis.

An UHPLC-Q Exactive system coupled with an HSS T3 chromatography column (100 mm × 2.1 mm i.d., 1.8 µm) was used to detect metabolites. The HPLC mobile phase was composed of 0.1% formic acid in water:acetonitrile (95:5, vol/vol) and 0.1% formic acid in acetonitrile: isopropanol:water (47.5:47.5:5, vol/vol). Mass spectrometer coupled with an ionization (ESI) source was operated based on a full scan of 70–1,050 *m/z*. The data acquisition was carried out using Data Dependent Acquisition mode.

### Data analysis

In this study, each treatment included three replications, and each experiment was repeated twice. The experimental data were expressed as mean ± standard deviation (SD). A statistically significant difference was conducted by one-way analysis of variance based on Duncan’s multiple range test at *P* < 0.05 using SPSS Statistics 26.0 (IBM Corporation, New York, USA). The sequencing data of transcriptome and metabolome analyses were performed via a one-stop online analytic platform, Majorbio Cloud (https://cloud.majorbio.com/).

## RESULTS

### Inhibition effects of the VOCs on *B. cinerea*

**Fig 1:**
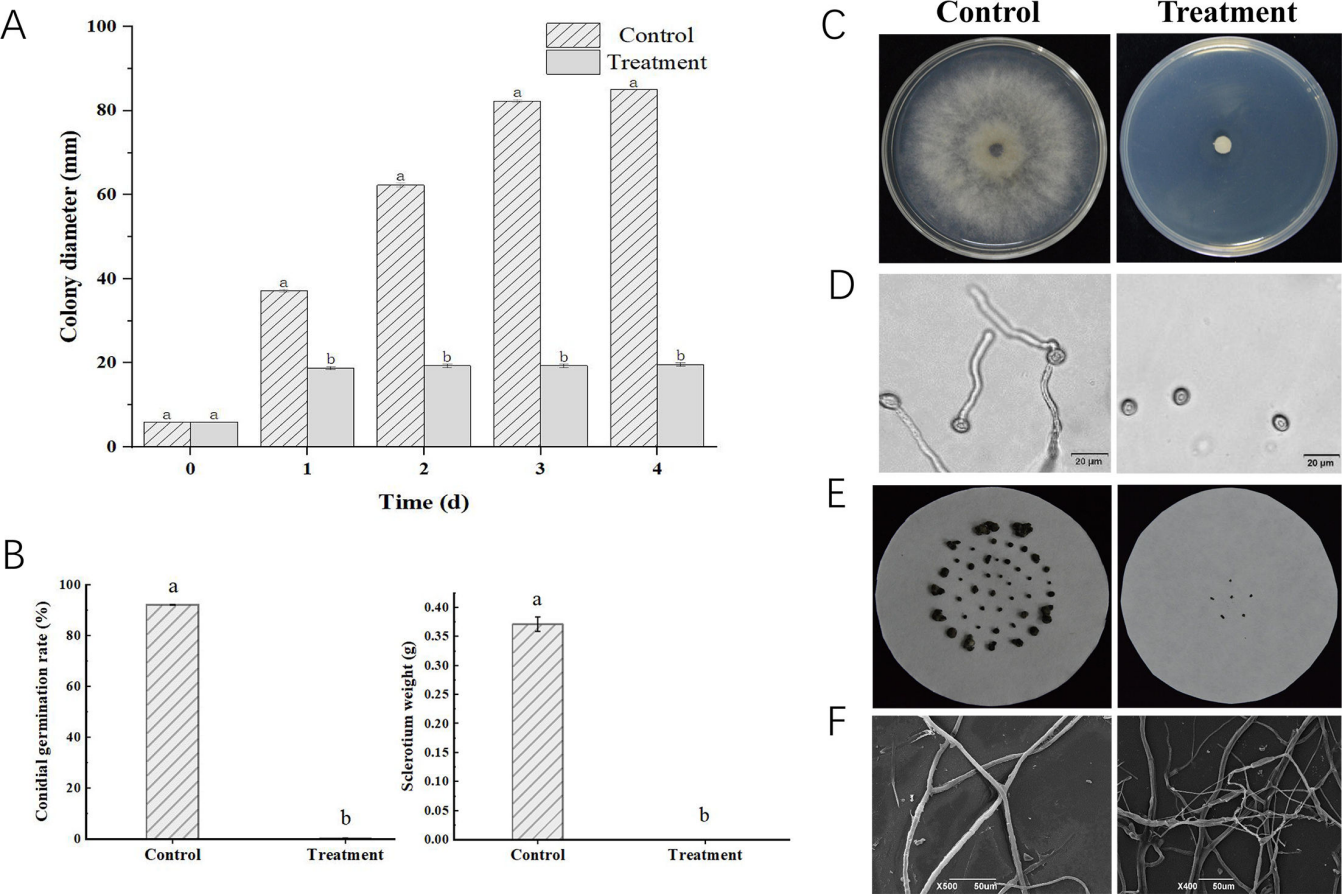
Antifungal effects of *P. chlororaphis* ZL3 VOCs on *B. cinerea*. (**A**) Effects of the VOCs on mycelial growth. (**B**) Effects of the VOCs on spore germination and sclerotium weight. (**C**) Mycelial growth of *B. cinerea* treated with the VOCs for 4 days. (**D**) Spore germination of *B. cinerea* treated with the VOCs for 6 h. (**E**) Sclerotium size and number of *B. cinerea* treated with the VOCs for 20 days. (**F**) Mycelial morphology of *B. cinerea* treated with the VOCs for 7 days. Each treatment included three replications, and each experiment was repeated twice. Values were expressed as mean ± standard deviation. Significant differences to control are marked with different letters via Duncan’s multiple range test (*P* < 0.05).

The inhibition effects of *P. chlororaphis* ZL3 VOCs on *B. cinerea* were shown in Fig. 1. Mycelial growth was significantly inhibited by the VOCs (*P* < 0.05), with the colony diameters ranging from 18.83 to 19.67 mm (Fig. 1A and C). Conidia germination rate of the VOCs treatment (0.31%) was significantly lower than the control (*P* < 0.05, Fig. 1B and D). Furthermore, the sclerotium numbers in VOCs treatment were only six, with a weight of 0.02 g, which exhibited a significant difference (*P* < 0.05, Fig. 1B and E). For further verification of the inhibition effect, *B. cinerea* mycelia were also observed by using SEM. In the control group, *B. cinerea* mycelia were normal and cylindrical hyphae. However, many distorted and collapsed fungal mycelia were found in the VOCs treatment (Fig. 1F).

### Effects of the VOCs on mycelia plasma membrane and protein concentration

**Fig 2:**
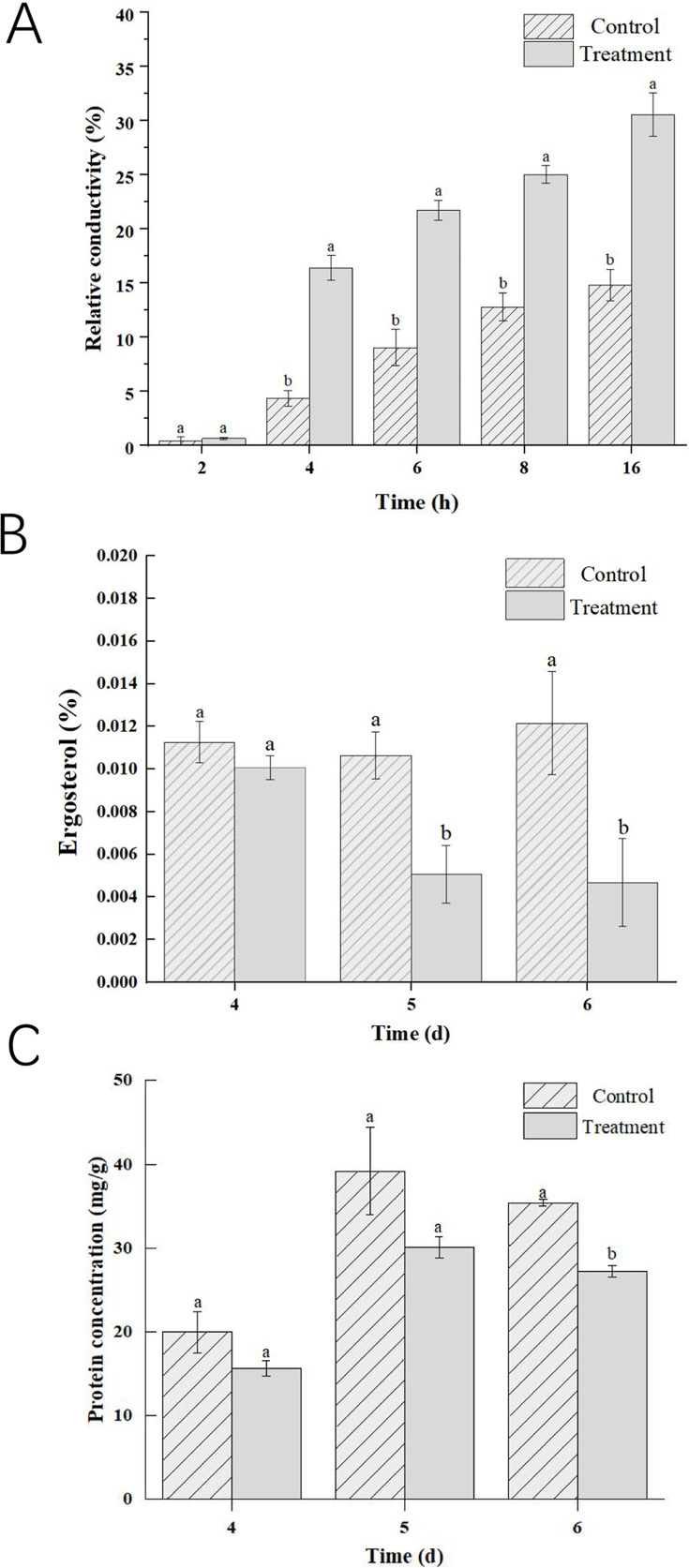
Effects of the VOCs released from *P. chlororaphis* ZL3 on the mycelial cell membrane, protein concentration. (**A**) Relative conductivity; (**B**) ergosterol relative content; (**C**) protein concentration. Each treatment included three replications, and each experiment was repeated twice. Values were presented as mean ± standard deviation. Significant differences to control are marked with different letters via Duncan’s multiple range test (*P* < 0.05).

Extracellular conductivity has been used to evaluate the extent of mycelial plasma membrane damage (29). The relative conductivities significantly increased in VOC treatments (*P* < 0.05, Fig. 2A). Within 5 and 6 days, the relative ergosterol contents were significantly lower than those in controls (*P* < 0.05). In particular, the relative ergosterol contents were significantly reduced by 61.43% (*P* < 0.05, Fig. 2B). After 6 days of inoculation, the protein concentrations in VOC treatments were significantly lower than those in control groups (*P* < 0.05, Fig. 2C).

### Effects of the VOCs on ATPase, SOD, CAT, PG, β-1, 3-GA, and CHI activities

**Fig 3:**
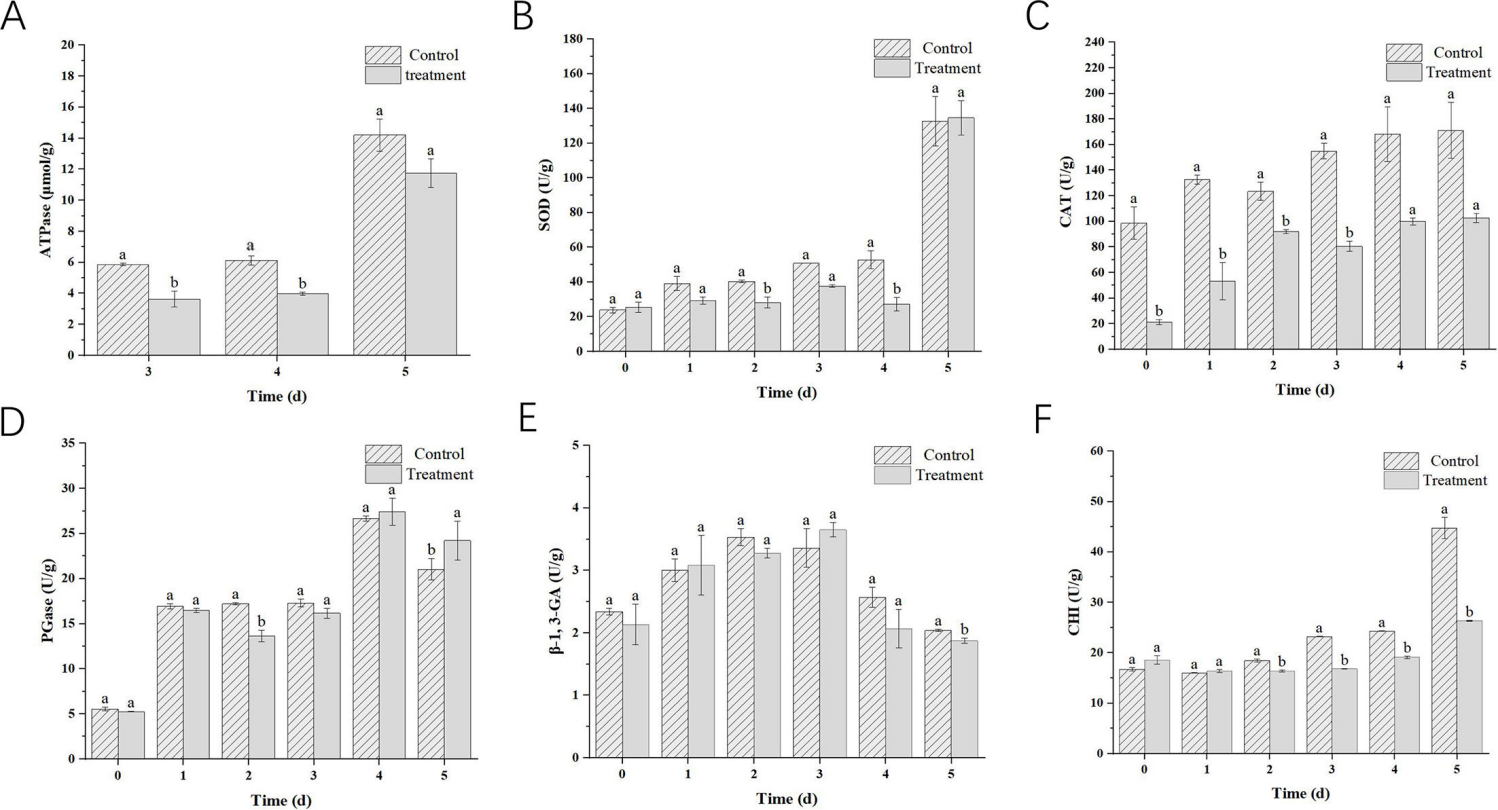
Effect of the VOCs released from *P. chlororaphis* ZL3 on enzymatic activities. (**A**) ATPase; (**B**) SOD; (**C**) CAT; (**D**) PG; (**E**) β-1,3-GA; and (**F**) CHI. Each treatment included three replications, and each experiment was repeated twice. Values were presented as mean ± standard deviation. Significant differences to control are marked with different letters via Duncan’s multiple range test (*P* < 0.05).

ATPase activities were significantly trended downward in VOCs treatments at 3 and 4 days after inoculation (*P* < 0.05, Fig. 3A). SOD and CAT activities were reduced in *B. cinerea* mycelia treated with the VOCs (Fig. 3B and C). In particular, the SOD activities significantly decreased to 48.3% at 4 days after treatment (*P* < 0.05, Fig. 3B). CAT activities significantly decreased during 0 and 3 days (*P* < 0.05, Fig. 3C). Most of the PG and β-1,3-GA activities exhibited no significant differences between VOCs treatment and control (Fig. 3D and E). Particularly, CHI activities in VOC treatments significantly decreased to 41.1% at 5 days after treatment (*P* < 0.05, Fig. 3F).

### Effects of the VOCs on DNA content and ROS accumulation

**Fig 4:**
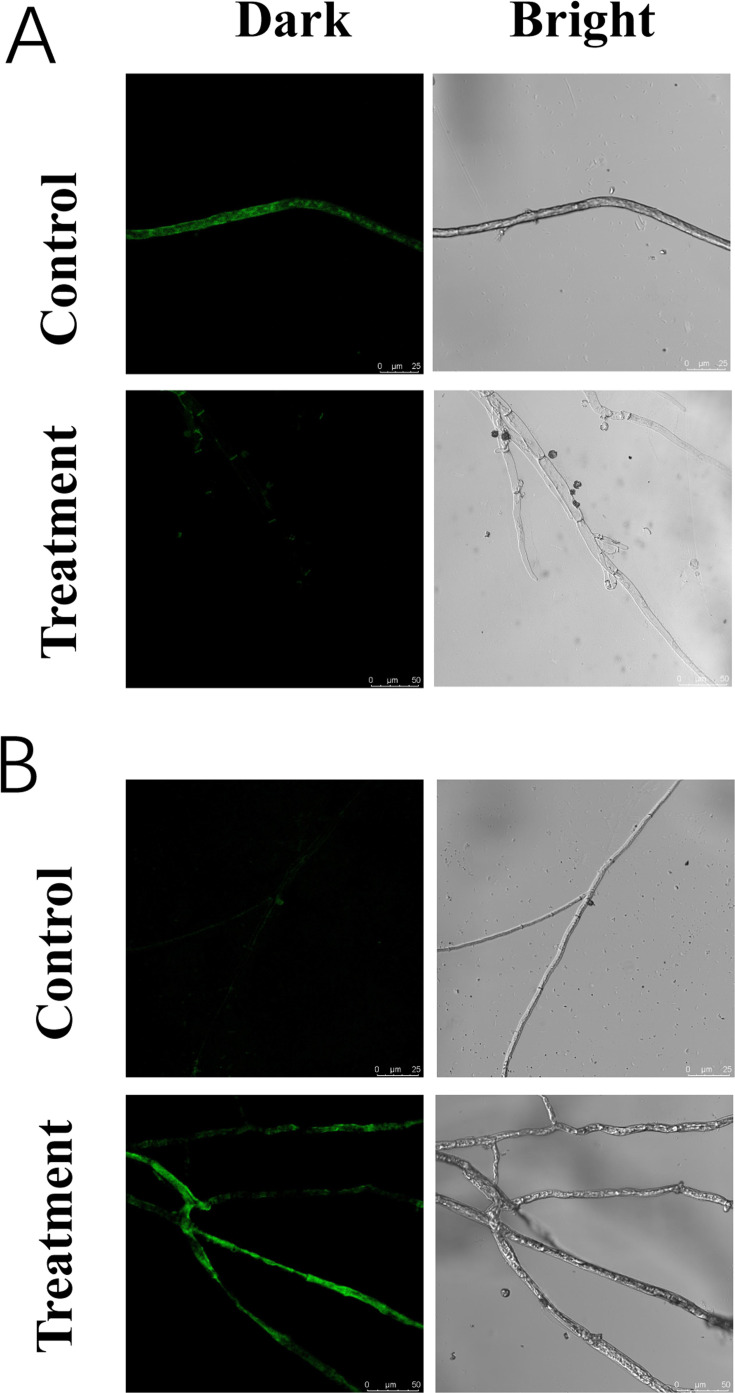
DNA content (**A**) and reactive oxygen species levels (**B**) in *B. cinerea* mycelia exposed to VOCs produced by *P. chlororaphis* ZL3 for 4 days.

DNA content and ROS accumulation were investigated using CLSM. The control mycelia exhibited intense green fluorescence (with the fluorescence intensity value of 143.7 ± 11.39), whereas that of the VOCs-treated mycelia was weak (53.4 ± 19.7, Fig. 4A), determining the VOCs could cause DNA damage. DCFH-DA staining found that the VOCs-treated mycelia had brighter green fluorescence (149.7 ± 13.99) than the control groups (48.7 ± 6.97, Fig. 4B), thereby indicating the VOCs could cause ROS accumulation.

### Transcriptome profiles in *B. cinerea* treated with *P. chlororaphis* ZL3 VOCs

Transcriptomic analysis was conducted to reveal the molecular variation in *B. cinerea* after treatment with *P. chlororaphis* ZL3 VOCs. The distribution and quality control of transcriptome sequencing data are summarized in Table S2. The average GC content was 46.31%. Q20 and Q30 were at least up to 98.31% and 92.86%, respectively. Total mapped reads of all the samples ranged from 90.36% to 93.53%. The scatter plot exhibited that there were 1,132 significantly up-regulated genes and 1,589 significantly down-regulated genes under the VOCs stress (Fig. S1A). A violin plot indicated the high expression levels of differential genes (Fig. S1B). Then, a clustering heatmap was generated to present expression levels for DEGs. Additionally, three treated samples were clustered in one clade and separated from the control samples, indicating the huge differences between the treatment and control groups (Fig. S1C).

GO functional annotation of DEGs was listed in Fig. S1D. As shown in Fig. S1E, a total of 645 DEGs were mainly annotated into several KEGG pathways, such as metabolism (accounted for 68.52%) and genetic information processing (accounted for 20.47%). These DEGs were enriched into 20 categories in the EggNOG database (Fig. S2). Most of the DEGs were enriched in carbohydrate transport and metabolism (215 genes), amino acid transport and metabolism (111 genes), secondary metabolites biosynthesis (110 genes), and translation, ribosomal structure, and biogenesis (105 genes). To validate the expression levels of transcriptomic sequencing, eight DEGs were used for RT-qPCR. The expression levels were consistent with those from transcriptomic sequencing, determining that transcriptomic sequencing data were highly reliable (Fig. S1F).

**Fig 5:**
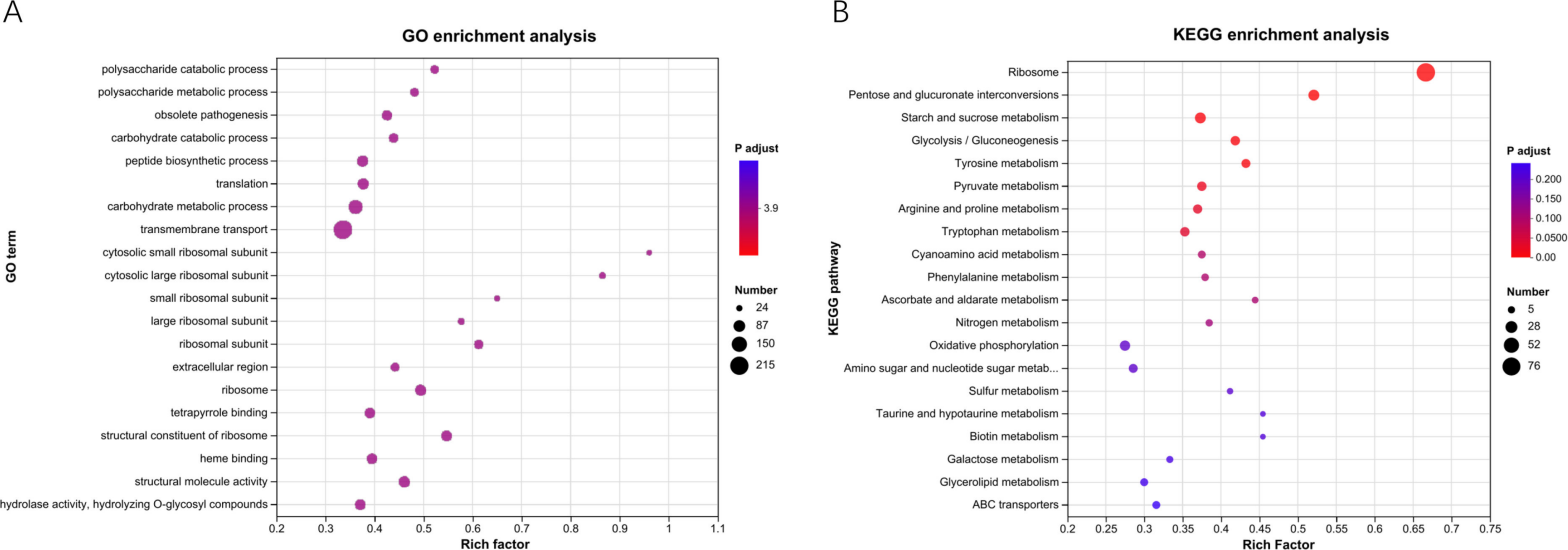
Top 20 terms for enrichment analyses of DEGs. (**A**) GO enrichment analysis; (**B**) KEGG enrichment analysis. The ordinate represents the enrichment terms, and the abscissa represents rich factor.

For a better understanding of the response of *B. cinerea* to the VOCs, GO and KEGG enrichment analyses were further conducted. Based on GO enrichment analysis, the DEGs were mainly enriched in polysaccharide catabolic process, polysaccharide metabolic process, and carbohydrate catabolic process (Fig. 5A). KEGG enrichment analysis indicated that the most enriched pathways were associated with pentose and glucuronate interconversions, starch and sucrose metabolism, and oxidative phosphorylation (Fig. 5B).

### Metabolomic analysis of *B. cinerea* treated with *P. chlororaphis* ZL3 VOCs

In partial least squares analysis (PLS-DA) scores plot with positive ion mode, component 1 and component 2 explained 66.3% and 5.58%, respectively (Fig. S3A). In the PLS-DA scores plot with negative ion mode, component 1 and component 2 explained 66.9% and 7.66%, respectively (Fig. S3C). R^2^ values were all above Q^2^ values, determining that the models were well fitted and highly predictable (Fig. S3B and D). Next, 569 differential metabolites were screened based on the criteria of VIP ≥1 and the *P* values <0.05 (Fig. S3E). Among these differential metabolites, 302 differential metabolites were obtained in positive ion mode (Fig. S3F), while 267 differential metabolites were obtained in negative ion mode (Fig. S3G). Whether in treatment or control groups, the relevant correlation values were all above 0.93 in the matrix, indicating the high consistency within each group (Fig. S3H).

**Fig 6:**
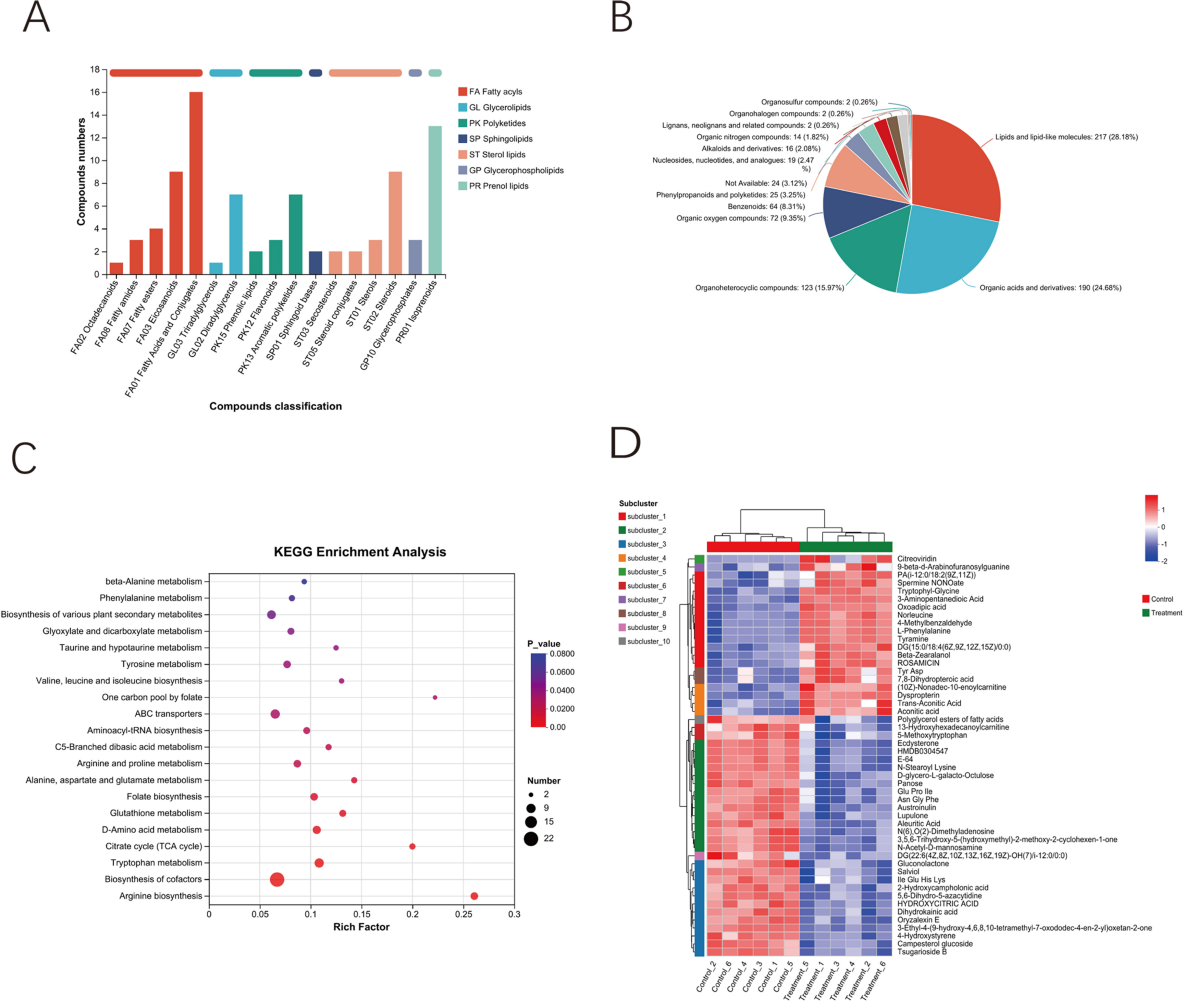
Analysis of differential metabolites after treatment with the VOCs. (**A**) KEGG compounds classification; (**B**) HMDB classifications; (**C**) KEGG enrichment analysis; (**D**) hierarchical cluster analysis for differential metabolites in different samples. Red and blue colors represent the high and low expression levels, respectively.

Subsequently, the differential metabolites were annotated into 17 KEGG compound classification, of which the most overrepresented compounds were fatty acids and conjugates, followed by isoprenoids, steroids, eicosanoids, diradylglycerols, and aromatic polyketides (Fig. 6A). Compared to the HMDB database, these differential metabolites were matched to 13 super classes (Fig. 6B). To reveal the significant metabolic pathways, these differential metabolites obtained were also annotated via KEGG enrichment analysis (Fig. 6C). Compared with the control groups, the most significant pathway was arginine biosynthesis, followed by biosynthesis of cofactors and tryptophan metabolism. In Fig. 6D, six replications, whether treatment or control groups, had obviously clustered in one clade. However, the samples between the treatment and control groups were dramatically separated into different clades, suggesting good repeatability of the tested samples.

### Integrated analysis of transcriptomic and metabonomic data

**Fig 7:**
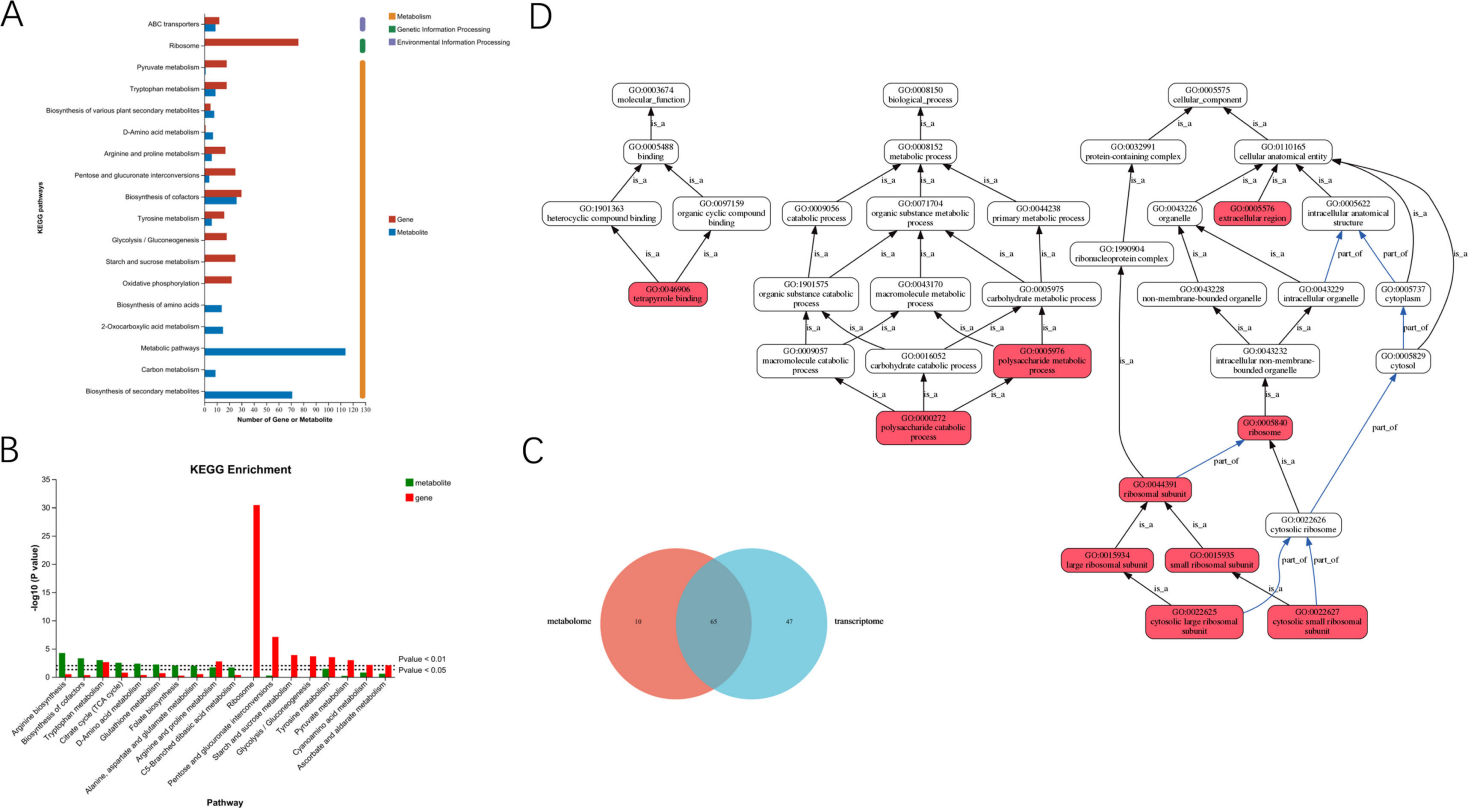
Relation analysis of DEGs and differential metabolites. (**A**) KEGG pathway annotation for DEGs and differential metabolites; (**B**) Venn diagram for KEGG pathway classification; (**C**) the *P* value histogram for KEGG enrichment analysis; and (**D**) directed acyclic graph of GO enrichment of DEGs.

The DEGs and differential metabolites were annotated into l12 and 75 KEGG pathways, respectively, of which 65 KEGG pathways were co-annotated by both DEGs and differential metabolites (Fig. 7C). The KEGG pathways were involved in metabolism, genetic information processing, and environmental information processing (Fig. 7A). Especially, biosynthesis of cofactors, pentose and glucuronate interconversions, tryptophan metabolism, ABC transporters, and arginine and proline metabolism were considered as the main co-annotated KEGG pathways (Fig. 7A). Moreover, natural metabolites, tryptophan metabolism, and arginine and proline metabolism were found to be the significantly enriched KEGG pathways, whether DEGs or differential metabolites (Fig. 7B). The DEGs were also illustrated as directed acyclic graphs in Fig. 7D, which determined the relationships of the GO terms.

## DISCUSSION

In this study, the VOCs from *P. chlororaphis* ZL3 were proven to have dramatic antifungal activity against mycelial growth and exhibit significant inhibition effects on conidia germination and sclerotium formation. In our previous work, the VOCs composition of *P. chlororaphis* ZL3 was investigated using gas chromatograph-mass spectrometry, and twenty-three compounds, including six alkanes, four aldehydes and ketones, four alcohols, four alkenes, three acids and esters, one aromatic compound, and one sulfur compound were identified (19). In fact, among these VOCs, ethyl 2-methylbutyrate, 3-methyl-1-butanol, and 2-ethylhexanol have been proven to exhibit high inhibitory activities against *B. cinerea* (9). Hexanal was also found to reduce infection of tomatoes by *B. cinerea* (35). Dimethyl disulfide exhibited a significant inhibitory effect on three poplar canker pathogens, *Cytospora chrysosperma*, *Phomopsis macrospora*, and *Fusicoccum aesculi* (36). Interestingly, ethyl 2-methylbutyrate, 3-methyl-1-butanol, 2-ethylhexanol, and dimethyl disulfide accounted for the main proportions of *P. chlororaphis* ZL3 VOCs, indicating the VOCs might play a crucial role in postharvest gray mold on Chinese cherry. Convincingly, our findings confirmed the excellent inhibitory activity of VOCs from *P. chlororaphis* ZL3 against *B. cinerea*. Under the stress of VOCs, *B. cinerea* hyphae exhibited obviously shrunken and distorted morphology, which is similar to the studies involved in the ultrastructural disruption of *B. cinerea* hyphae in the treatment with the microbial VOCs from *Bacillus subtilis* (37) and *B. siamensis* (9). To our best knowledge, in this work, the antifungal activities of VOCs emitted from *P. chlororaphis* ZL3 were comprehensively investigated for the first time. The findings indicated that *P. chlororaphis* ZL3 VOCs could induce significant metabolic changes in *B. cinerea* and therefore prominently disrupt the hyphae cells, thus markedly suppressing the hyphae development, conidia germination, and sclerotium formation of *B. cinerea*.

Generally, numerous previous researches have focused on the biocontrol mechanisms of microbial VOCs against plant pathogens, mainly including the pathogen suppression, antifungal composition components, and induced systemic resistance to host pathogens (8, 38–40). Previous studies proved that microbial VOCs could affect the substance metabolism in plant pathogens (8, 23, 38). Nevertheless, only a few researchers have interpreted the biocontrol mechanisms of the microbial VOCs from *Pseudomona*s species against *B. cinerea* through physiological and transcriptomic analysis (8). In this work, we attempted to explain the antifungal molecular mechanism through biophysical, biochemical, transcriptomic, and metabolomic analyses.

The cell wall and cell membrane are essential for fungal cells and could be helpful to maintain the cell shape through increasing mechanical resistance (23). The fungal cell wall is essential for the growth and survival of fungi and is usually considered an important target for antifungal compounds (29). β-1,3-glucan and chitin are both the major macromolecules of the cell wall and are involved in cell-wall formation (23, 39). β-1,3-GA could break down β-1,3-glucan, while CHI could catalyze chitin hydrolysis and further influence the stability and integrity of fungal cells (23, 39). In this study, CHI activity demonstrated significant differences from 2 to 5 days, while β-1,3-GA activity always exhibited no significant differences between treatment and control groups. Therefore, CHI has the potential to target the cell walls of *B. cinerea*.

Cell membranes are of importance for cell integrity and material energy metabolism in fungi (41). As a crucial component in the membrane, ergosterol could be involved in membrane integrity and fluidity (8, 29). Many present studies have validated that ergosterol contents significantly decreased after treatment with microbial VOCs (8, 18, 29). Similarly, in our study, *P. chlororaphis* ZL3 VOCs could reduce ergosterol content in *B. cinerea*, ultimately damaging the membrane integrity. The extracellular conductivities were used to reflect the extent of mycelial plasma membrane damage (29). Interestingly, the extracellular conductivities were found to significantly increase in VOC treatments. Similar to our results, Duan et al. (29) reported that carvacrol treatment can increase extracellular conductivity. Thus, our studies also showed that *P. chlororaphis* ZL3 VOCs might damage the mycelial plasma membrane.

Mitochondria could play the core role in cellular energy metabolism and be responsible for 90% of the energy to fulfill physiological functions (42). ROS, highly reactive molecules, are usually accumulated inside the cell mitochondria under adverse conditions that could further lead to cellular damage to lipids, DNA, and proteins (42, 43). Some studies suggested that the VOCs produced from biocontrol microorganisms, such as *Pseudomonas fluorescens*, *Aureobasidium pullulans,* and *Corrallococcus* sp., could result in ROS accumulation (8, 44, 45). In this study, excessive accumulation of ROS was also found in *B. cinerea* treated with *P. chlororaphis* ZL3 VOCs. Moreover, compared to the control groups, the contents of DNA and protein both decreased remarkably in the treatments. Additionally, the ROS accumulation can lead to the abnormality of mitochondrial function (8). As an aerobic microorganism, *B. cinerea* is required to derive energy through mitochondrial respiratory function (46). ATPase is crucially important for the respiration and energy metabolism of microorganisms. In this experiment, ATPase activities of the treatments were found to be much lower than those of the controls, which is in agreement with the experimental data reported by Yue et al. (8). The findings have shown that *P. chlororaphis* ZL3 VOCs could disturb mitochondrial respiration and further adversely affect the cellular energy metabolism.

Fungal cells are armed with a mass of antioxidant defense mechanisms to avoid the damage of oxidative accumulation to cells (47). Antioxidant enzymes, such as SOD and CAT, were usually considered as biomarkers to reflect oxidative stress levels in cells and could play a very important role in the defense mechanisms (29, 48). Duan et al. (29) reported that the SOD and CAT activities were significantly reduced in *Aspergillus flavus* mycelia exposed to carvacrol. In our work, SOD and CAT activities were also found to be significantly decreased after treatment with *P. chlororaphis* ZL3 VOCs, indicating that *P. chlororaphis* ZL3 VOCs could reduce the antioxidation ability of *B. cinerea*.

To further disclose the molecular mechanism, the expression levels of genes involved in the inhibition effects were explored in this study. After treatment with *P. chlororaphis* ZL3 VOCs, a good deal of DEGs participated in the membrane part, membrane transport, and lipid metabolism, which might significantly affect the cell membranes’ structure and function. Particularly, 1382 DEGs were associated with membrane and part membrane transport, implying that the cell membrane might serve as a key target for the inhibition of *B. cinerea*. Energy metabolism is of great importance for fungal pathogens and provides a basis for fungal growth and multiplication (23). Several antifungal compounds can destroy the energy metabolism and then inhibit the fungal development (23, 49). Similarly, in this study, 84 DEGs, such as *Bcfhg1*, *Bcboa5*, and *Bcpck1,* were found to be associated with energy production and conversion. Through KEGG enrichment analysis, oxidative phosphorylation was shown to be one of the top KEGG pathways, indicating the abnormal oxidative phosphorylation in mitochondria. We also found that the DEGs were involved in the KEGG pathways of mitophagy, endocytosis, and autophagy. Autophagy homeostasis, considered a new biocontrol site, might be crucial for fungal survival and competition (18, 50).

Metabolomic analysis was performed to interpret the function of differential metabolites. In this study, the differential metabolites, including lipids and lipid-like molecules, and organic acids and derivatives, might be involved in the cell wall strength and antioxidant stress (7). The KEGG compound classification showed that the metabolites were mainly annotated into fatty acids and conjugates. Fatty acids are known as a preponderance component of fungal membranes and play vital roles in fungal life, including energy stores and post-translational modifications of membrane-bound proteins (38). Fatty acids have also been reported to be associated with fungal sporulation and mycelial growth (51, 52). Amino acid metabolism could contribute to a nutrient source for fungi and become a novel target for antifungal action (53). Of these metabolism processes, amino acid metabolism was found to be the significantly enriched KEGG pathway, indicating that *P. chlororaphis* ZL3 VOCs might result in the imbalance of metabolism progress, especially in amino acid metabolism, and ultimately inhibit the development of *B. cinerea*.

### Conclusion

This work found that *P. chlororaphis* ZL3 VOCs had excellent antifungal effects on mycelial growth, conidia germination, and sclerotium formation and severely damaged the morphology of *B. cinerea*. The action mechanisms of *P. chlororaphis* ZL3 VOCs against *B. cinerea* were comprehensively determined through transcriptomic, metabolomic, and biophysical analysis. In general, the VOCs could enhance cell membrane permeability, destroy cell membrane integrity, disturb mitochondrial respiration, and further adversely interfere with the energy metabolism via ROS accumulation and ATPase activity reduction. The VOCs from *P. chlororaphis* ZL3 could also reduce the antioxidation ability of *B. cinerea*. Transcriptomic and metabolomic data were further supported by our biophysical findings. A good deal of DEGs was closely involved in membrane components, lipid metabolism, membrane transport, and energy metabolism. The differential metabolites were mainly enriched in amino acid metabolism, carbohydrate metabolism, and lipid metabolism. Although more work should be done to reveal the antifungal components of VOCs and the relationship of different antifungal VOCs, this work would give a new perspective for the action mechanisms of microbial VOCs to *B. cinerea* and will be helpful for further development of new biological fumigation.

## Data Availability

All data are included in the article and its supplemental material. The raw RNA-Seq data used in this study were deposited in GenBank under BioProject PRJNA1099710.

## SUPPLEMENTAL MATERIAL

Fig. S1 spectrum.02706-25-s0001.pdf Transcriptome profiles.

Fig. S2 spectrum.02706-25-s0002.pdf EggNOG functional classification.

Fig. S3 spectrum.02706-25-s0003.pdf Quality control of metabolomics profiles.

Supplemental material spectrum.02706-25-s0004.docx Legends for Supplemental figures.

Supplemental tables spectrum.02706-25-s0005.docx Tables S1 and S2.

ASM does not own the copyrights to Supplemental Material that may be linked to, or accessed through, an article. The authors have granted ASM a non-exclusive, world-wide license to publish the Supplemental Material files. Please contact the corresponding author directly for reuse.
