# Supplementary figures and images for: Multifaceted antifungal mechanisms of volatile organic compounds emitted from Pseudomonas chlororaphis ZL3 against Botrytis cinerea
Source: Microbiol Spectr. 2025 Nov 24;14(1):e02706-25. doi: 10.1128/spectrum.02706-25 (PMC12772388; doi:10.1128/spectrum.02706-25)

A

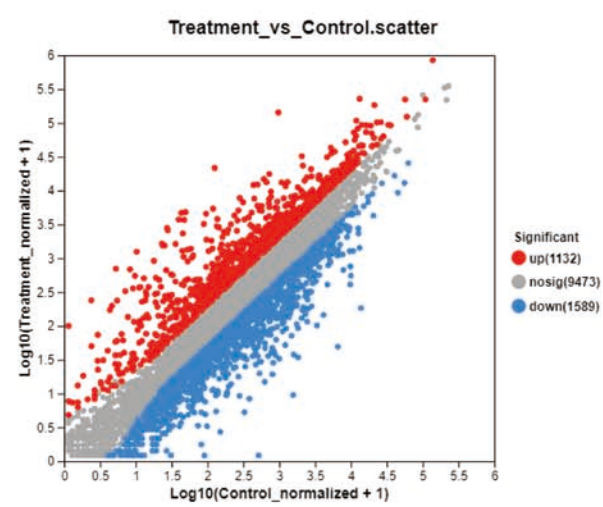

B

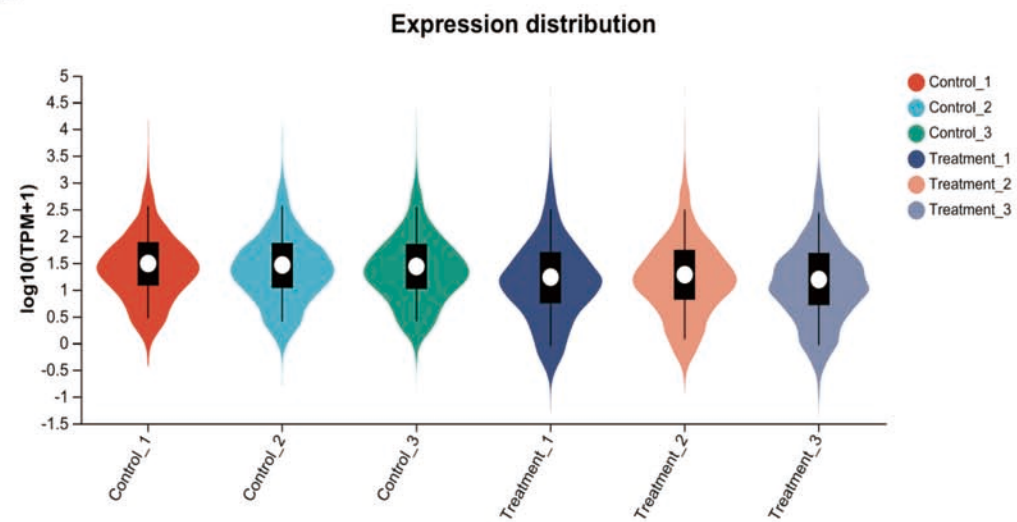

C

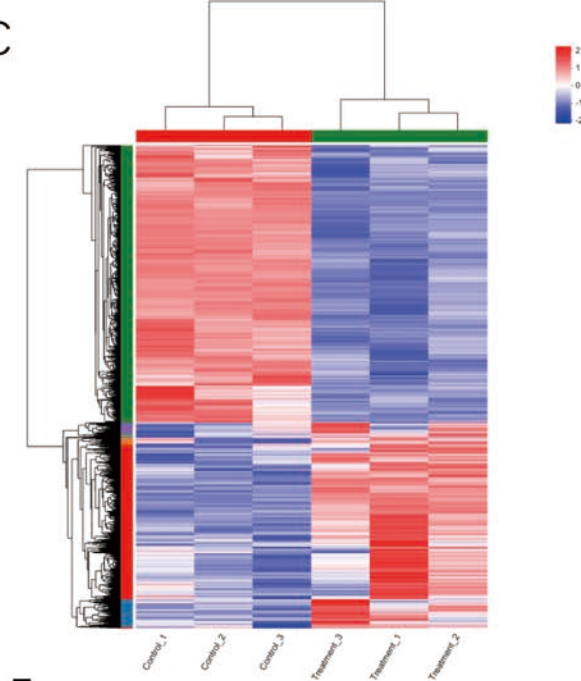

D

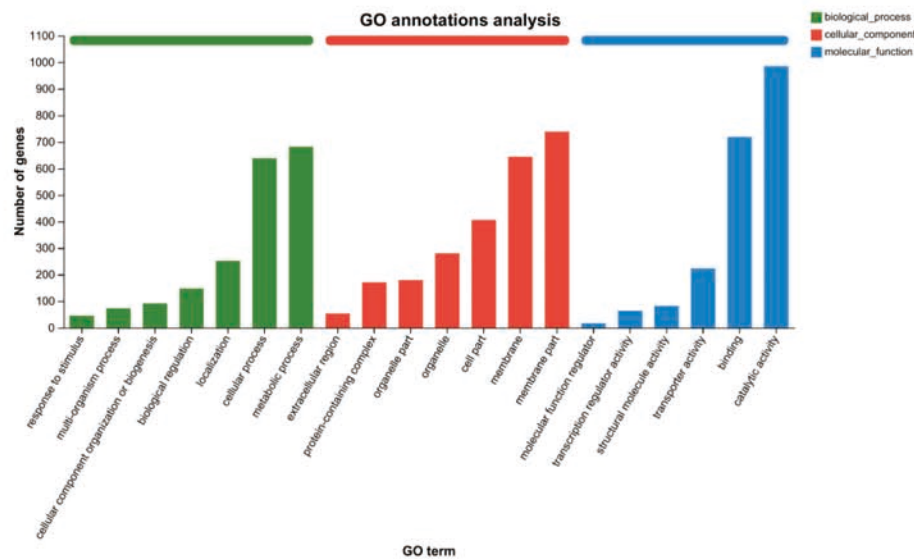

E

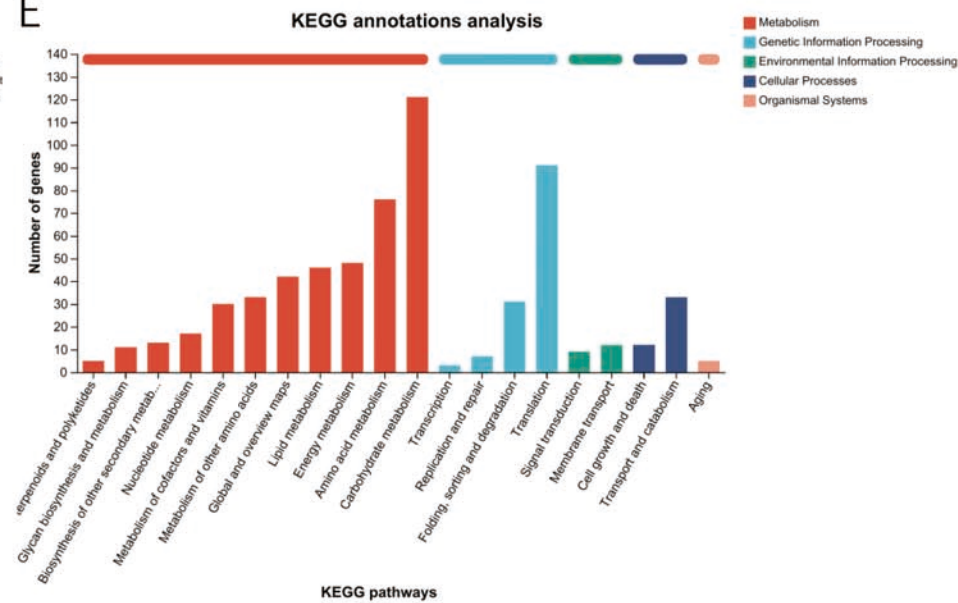

F

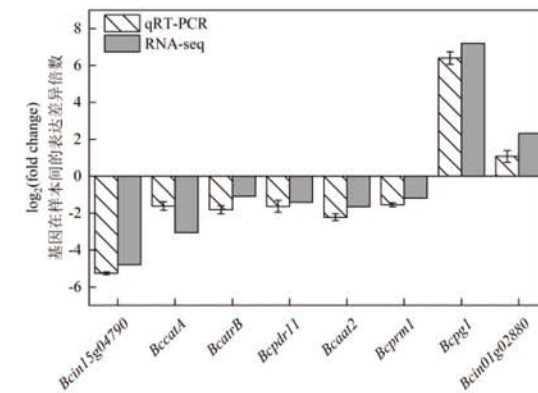

Supplement: Fig. S1 — Transcriptome profiles. [file spectrum.02706-25-s0001.pdf]

# EggNOG classification

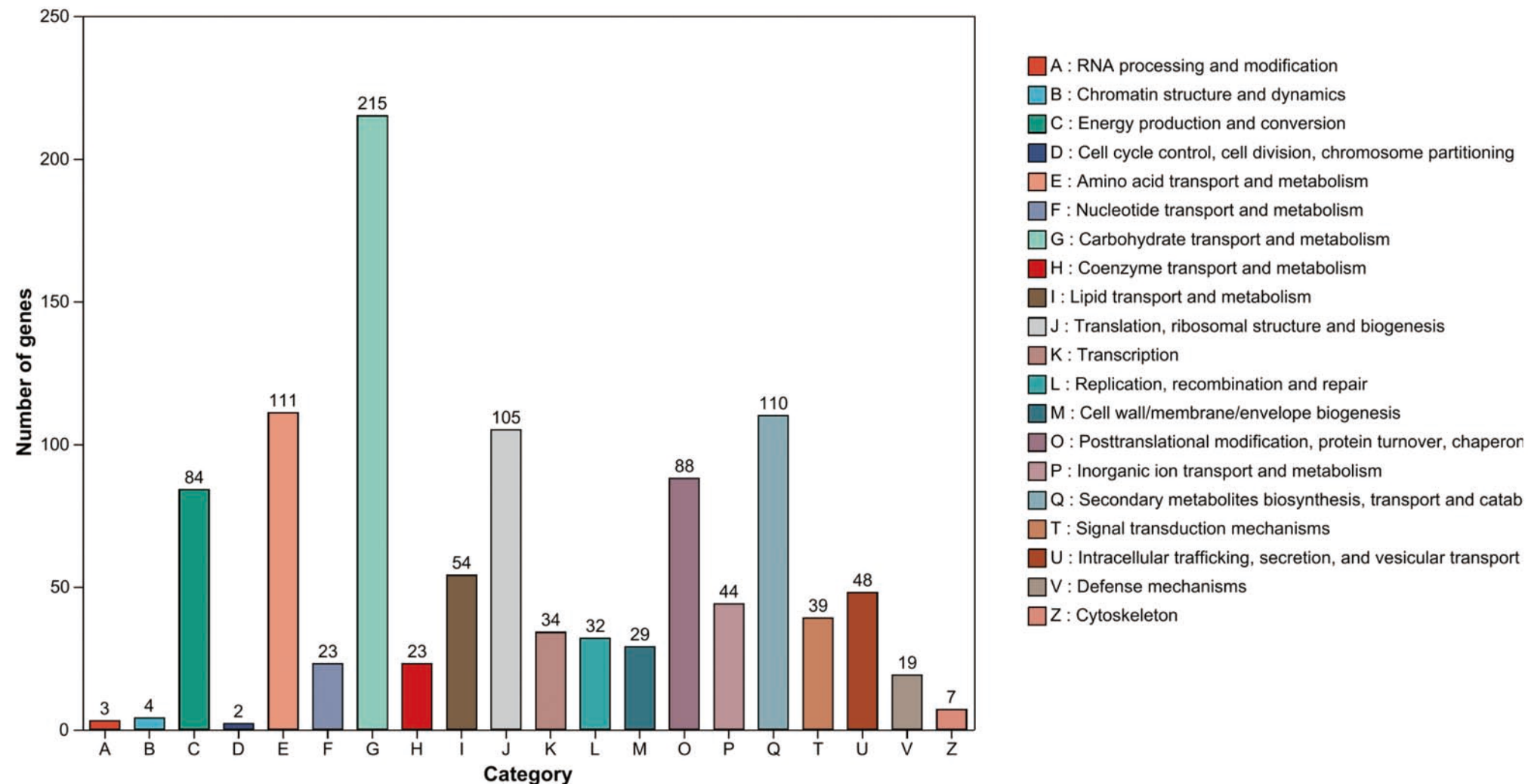

Supplement: Fig. S2 — EggNOG functional classification. [file spectrum.02706-25-s0002.pdf]

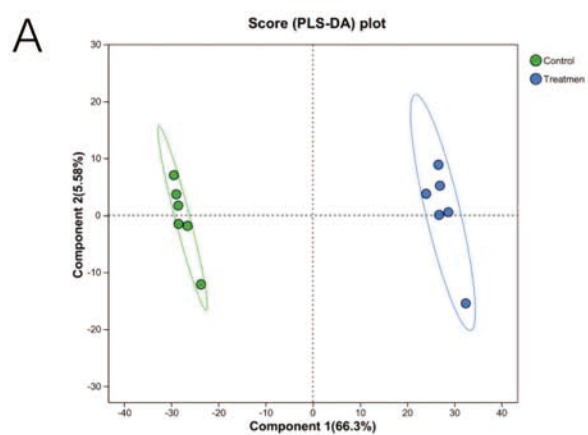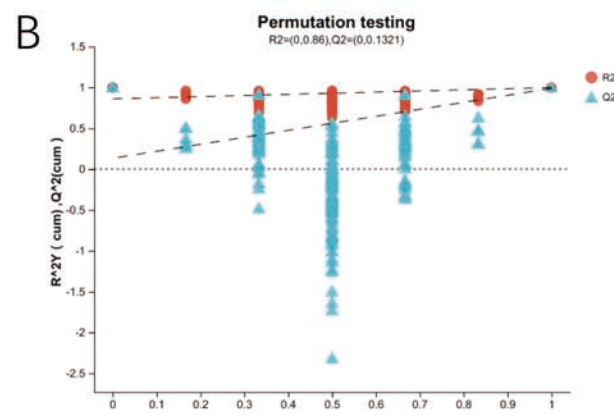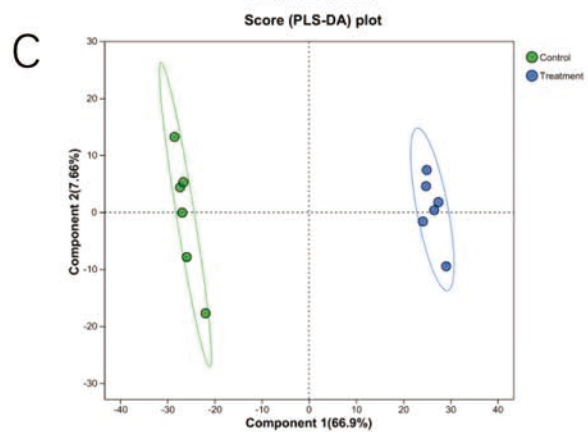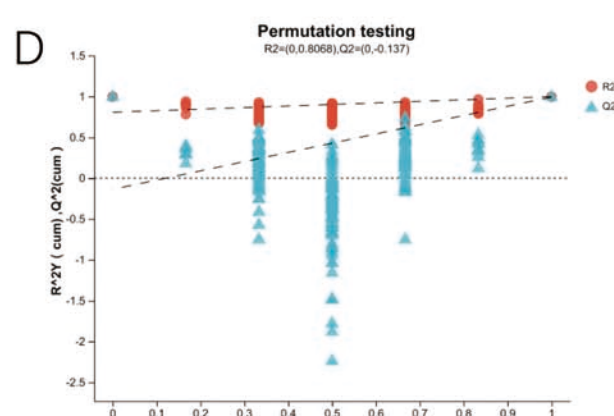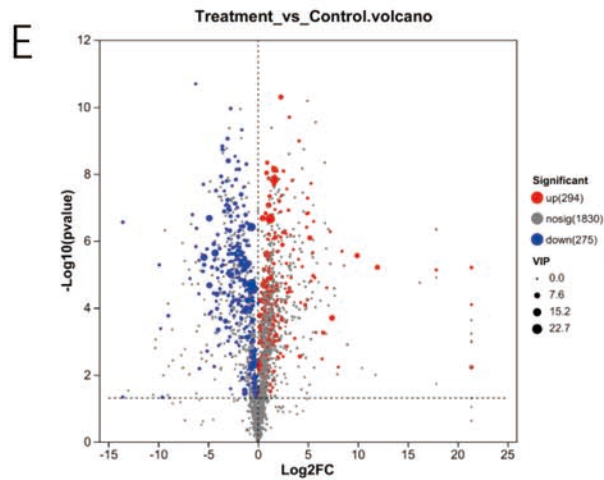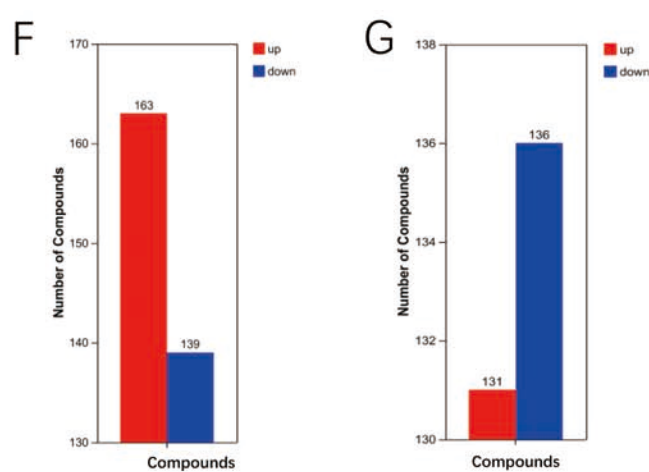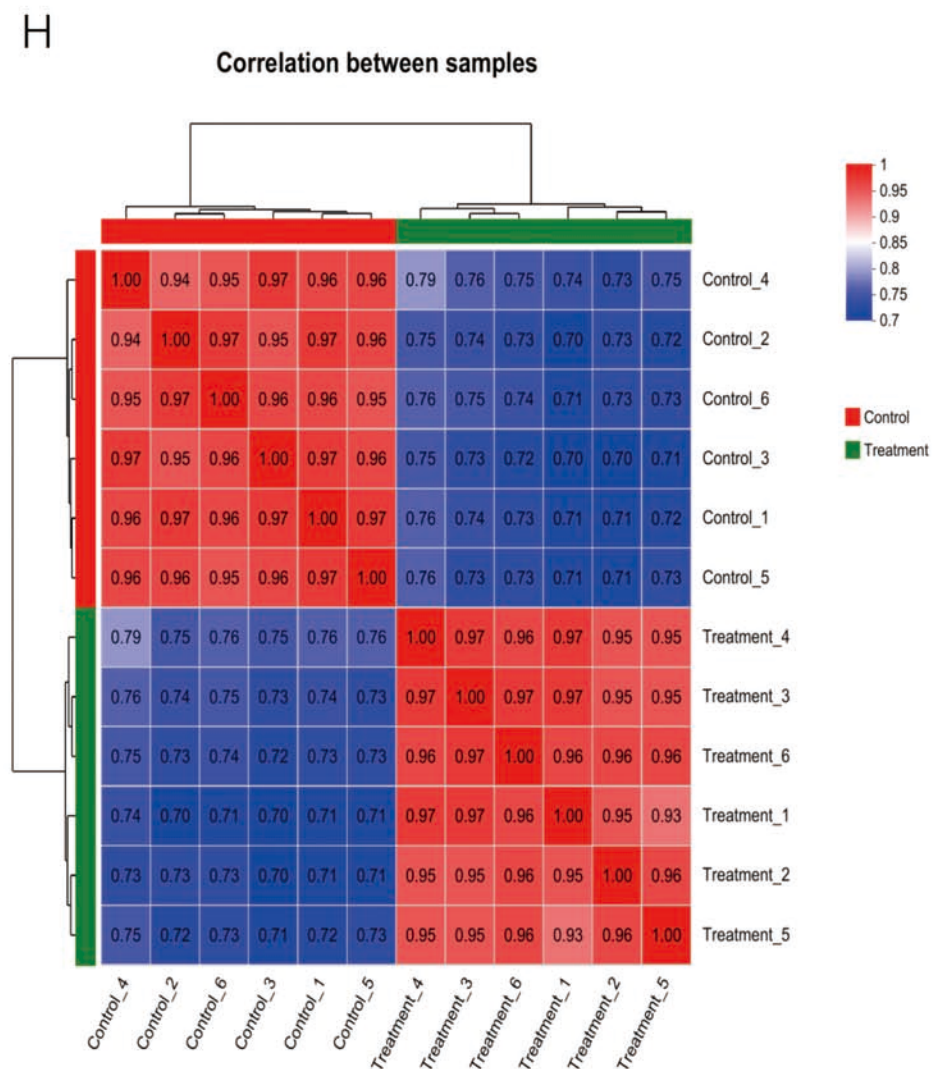

Supplement: Fig. S3 — Quality control of metabolomics profiles. [file spectrum.02706-25-s0003.pdf]
