# Supplementary material for: Multifaceted antifungal mechanisms of volatile organic compounds emitted from Pseudomonas chlororaphis ZL3 against Botrytis cinerea
Source: Microbiol Spectr. 2025 Nov 24;14(1):e02706-25. doi: 10.1128/spectrum.02706-25 (PMC12772388; doi:10.1128/spectrum.02706-25)
Supplement: Supplemental material — Legends for Supplemental figures. [file spectrum.02706-25-s0004.docx]

**Supplemental Figures legends:**

**Fig. S1.** Transcriptome profiles in *B. cinerea* treated with *Pseudomonas chlororaphis* ZL3 VOCs. (A) The scatter plot of differential gene expression; (B) The violin plot for the distribution of DEGs; (C) Heat map of gene expression abundance in treatment and control groups; (D) GO functional annotation analysis; (E) KEGG pathway annotation analysis; (F) Relative expression levels of eight DEGs for RNA-Seq and RT-qPCR.

Fig. S1 will be placed in Line 256, Page 11.

**Fig. S2.** EggNOG functional classification of the DEGs.

Fig. S2 will be placed in Line 264, Page 12.

**Fig. S3.** Quality control of metabolomics profiles in *B. cinerea* with the VOCs treatments. (A) PLS-DA scores plot in positive ion mode; (B) PLS-DA permutation test in positive ion mode; (C) PLS-DA scores plot in negative ion mode; (D) PLS-DA permutation test in negative ion mode; (E) Volcano map of differential metabolites; (F) The numbers of differential metabolites in positive ion mode; (G) The numbers of differential metabolites in negative ion mode; (H) Correlation of the different samples between treatment and control groups.

Fig. S3 will be placed in Line 279, Page 12.
