# Supplementary material for: Multifaceted antifungal mechanisms of volatile organic compounds emitted from Pseudomonas chlororaphis ZL3 against Botrytis cinerea
Source: Microbiol Spectr. 2025 Nov 24;14(1):e02706-25. doi: 10.1128/spectrum.02706-25 (PMC12772388; doi:10.1128/spectrum.02706-25)
Supplement: Supplemental tables — Tables S1 and S2. [file spectrum.02706-25-s0005.docx]

**Supplemental Table captions:**

**Table S1** The primer sets used for qRT-PCR.

Table S1 will be placed in Line 187, Page 9.

**Table S2** Distribution and quality control of the RNA-seq data.

Table S2 will be placed in Line 252, Page 11.

Table S1 The primer sets used for qRT-PCR

| Gene name | | Forward primers (5′-3′) | Reverse primers (5′-3′) |
| --- | --- | --- | --- |
| *Actin* | ATCTTGGCTGAGCGTGGTTA | ACTGGCGGTTTGGATTTCTT |  |
| *Bcin15g04790* | CTGCTATCACCACCCAGCAA | TGCGGGTGATCCACTCTTTC |  |
| *BccatA* | AAGCCGCCCTTTCATCTTGTAACTC | GCAGCACGGTCACGATTGTCTC |  |
| *BcatrB* | TGTCTTTCTGCGGTGTCCTC | AAGACGAGGAGAGAGCCCAT |  |
| *Bcpdr11* | TTCTCGGCGATGATGAGACG | ATTTAAGCCCCAGCAGGTCC |  |
| *Bcaat2* | TGTCGGAGTCCTTCACGTTG | TCAGTTGAGTGAGTGACGGC |  |
| *Bcprm1* | GACTCAAAATCGCGGGCATC | AGGAAATTGTCGTCTGCGGT |  |
| *Bcpg1* | GATGTTGGTTCCTCCAGCGA | CGGAGACAGTGTTGTCGGAA |  |
| *Bcin01g02880* | GGATCGCCTATGCTTGTGGT | AGAGGAGCAGCGTCCTTAGA |  |

Table S2 Distribution and quality control of the RNA-seq data

| Samples | Raw reads | Clean reads | Mapped reads | Mapped rate (%) | Error rate (%) | Q20 (%) | Q30 (%) | GC content (%) |
| --- | --- | --- | --- | --- | --- | --- | --- | --- |
| Control_1 | 42270324 | 41882124 | 38694906 | 92.39 | 0.02 | 98.15 | 94.33 | 45.92 |
| Control_2 | 43151652 | 42831790 | 40059465 | 93.53 | 0.03 | 98.01 | 93.98 | 45.98 |
| Control_3 | 44861480 | 44470722 | 41245848 | 92.75 | 0.03 | 98.03 | 94.04 | 46.00 |
| Treatment_1 | 45832640 | 45532878 | 41143810 | 90.36 | 0.02 | 98.30 | 94.73 | 46.75 |
| Treatment_2 | 45318156 | 45016194 | 41137097 | 91.38 | 0.02 | 98.21 | 94.46 | 46.40 |
| Treatment_3 | 41218600 | 40887632 | 37919260 | 92.74 | 0.03 | 98.06 | 94.08 | 46.78 |
